# Efficacy of heads‐up CPR compared to supine CPR positions: Systematic review and meta‐analysis

**DOI:** 10.1002/hsr2.644

**Published:** 2022-05-24

**Authors:** Joseph Varney, Karam R. Motawea, Mostafa R. Mostafa, Yossef H. AbdelQadir, Merna Aboelenein, Omneya A. Kandil, Nancy Ibrahim, Hashim T. Hashim, Kimberly Murry, Garrett Jackson, Jaffer Shah, Maty Boury, Ahmed K. Awad, Priya Patel, Dina M. Awad, Samah S. Rozan, Nesreen E. Talat

**Affiliations:** ^1^ School of Medicine American University of the Caribbean Philipsburg Sint Maarten (Dutch Part); ^2^ Faculty of Medicine Alexandria University Alexandria Egypt; ^3^ Rochester Regional Health/Unity Hospital Rochester New York USA; ^4^ College of Medicine University of Baghdad Baghdad Iraq; ^5^ Barry University Palm Beach Gardens USA; ^6^ Kateb University Medical Research Center Kateb University Kabul Afghanistan; ^7^ New York State Department of Health New York USA; ^8^ Faculty of Medicine Ain‐Shams University Cairo Egypt

**Keywords:** cardiac arrest, cardiopulmonary resuscitation, emergency medical services, heads‐up CPR

## Abstract

**Background and Aim:**

Cardiopulmonary resuscitation (CPR) in full‐coded patients requires effective chest compressions with minimal interruptions to maintain adequate perfusion to the brain and other vital organs. Many novel approaches have been proposed to attain better organ perfusion compared to traditional CPR techniques. The purpose of this review is to investigate the safety and efficacy of heads‐up CPR versus supine CPR.

**Methods:**

We searched PubMed Central, SCOPUS, Web of Science, and Cochrane databases from 1990 to February 2021. After the full‐text screening of 40 eligible studies, only seven studies were eligible for our meta‐analysis. We used the RevMan software (5.4) to perform the meta‐analysis.

**Results:**

In survival outcome, the pooled analysis between heads‐up and supine CPR was (risk ratio = 0.98, 95% confidence interval [CI] = 0.17–5.68, *p* = 0.98). The pooled analyses between heads‐up CPR and supine CPR in cerebral flow, cerebral perfusion pressure and coronary perfusion pressure outcomes, were (mean difference [MD] = 0.10, 95% CI = 0.03–0.17, *p* = 0.003), (MD = 12.28, 95% CI = 5.92–18.64], *p* = 0.0002), and (MD = 8.43, 95% CI = 2.71–14.14, *p* = 0.004), respectively. After doing a subgroup analysis, cerebral perfusion was found to increase during heads‐up CPR compared with supine CPR at 6 min CPR duration and 18 to 20 min CPR duration as well.

**Conclusion:**

Our study suggests that heads‐up CPR is associated with better cerebral and coronary perfusion compared to the conventional supine technique in pigs' models. However, more research is warranted to investigate the safety and efficacy of the heads‐up technique on human beings and to determine the best angle for optimization of the technique results.

## 1 INTRODUCTION

Cardiopulmonary resuscitation (CPR) requires effective and timely chest compressions with minimal interruptions to maintain adequate organ perfusion and hence prevent irreversible organ damage. In 2019, the American Heart Association estimated around 356,000 cases of out‐of‐hospital cardiac arrest (OHCA) as well as 209,000 cases of in‐hospital cardiac arrest. Unfortunately, only 8.4% of OHCA exhibited satisfying neurological outcomes upon hospital discharge.^1^ In successfully resuscitated patients, anoxic brain injury served as the most common cause of death after an OHCA. Therefore, an effective CPR technique is critical to improve cerebral blood flow and reduce irreversible brain damage in post‐cardiac arrest patients.^2^

Compared to traditional CPR techniques, novel approaches were designed to ensure continued blood flow to the brain and vital organs.^3^ They entail head and chest elevation to enhance blood return to the heart by gravity. This would seem counterintuitive with the blood being taken away from the brain. When considering passive leg raising techniques used to increase preload to the heart, it would make sense that elevating the head would work along the same lines. One significant difference between these two approaches is that blood pooled in the brain causes pressure due to the skull compartment. Intracranial pressure elevation can cause brain damage, making brain venous runoff a critical consideration.^3^

Patients were shown to benefit from being in an elevated position during transport and CPR. Recent research suggests reducing stretcher length could allow for manipulation of the patient's body and maximize the delivery of high‐quality CPR.^4^ Several emergency medical service (EMS) systems in the United States have adopted head elevation techniques among other advances and resulted in raised resuscitation and survival rates for all arrested patients.^5^ A very good EMS system example is the one adopted in Palm Beach County, Florida which raised resuscitation rates for all patients from 18% to 34% and from 23% to more than 44% among those with ventricular fibrillation (VF)/ventricular tachycardia arrests.^5^, ^6^, ^7^ We aim to provide a thorough review of the utility and feasibility of the heads‐up CPR technique as well as the necessary training and equipment to perform successful CPR.

## 2 METHODS

We conducted this systematic review according to the guidelines of the Cochrane handbook for systematic reviews and meta‐analysis. We also followed the criteria of the preferred reporting items of systematic reviews and meta‐analysis (PRISMA Statement) during this review preparation.

### 2.1 Search strategy

We searched PubMed Central, SCOPUS, web of science, and Cochrane databases using the following (Resuscitation, Cardiopulmonary)) OR (Cardio‐Pulmonary Resuscitation)) OR (Cardio Pulmonary Resuscitation)) OR (Resuscitation, Cardio‐Pulmonary)) AND ((patient positioning) OR (elevated head) OR (tilted head) OR (head tilt) OR (heads up) OR (elevated chest*) OR (elevated thorax) till February 2021. A manual search was performed using the references of the included articles. All the results were added to covidence platform for further screening.

### 2.2 Eligibility criteria and study selection

We included in the meta‐analysis only trials that compare heads‐up CPR with supine position CPR in cardiac arrest on pig subjects because no studies on humans compare heads‐up CPR with supine position CPR in cardiac arrest—published and excluded any review, case report, systemic review, and the studies not available in English. Reviewers independently screened the titles and abstracts of the trials, followed by the full‐text screening for confirmation to include the articles in our study.

### 2.3 Data extraction

Authors were divided into two groups to extract data from the included studies: (1) summary including study design, groups, and sample size (name and number of each group), results and (2) outcomes including Coronary perfusion pressure (mmHg), cerebral perfusion pressure (mmHg), cerebral flow (ml/min), coronary blood flow (ml/min), systolic, and diastolic aortic pressure and survival.

### 2.4 Risk of bias assessment

We used the Cochrane Risk of Bias tool provided in the Cochrane handbook for systematic reviews of interventions. The domains included were^1^: Random sequence generation (selection bias).^2^ Allocation concealment (selection bias).^3^ Blinding of participants and personnel (performance bias).^4^ Outcomes assessment (detection bias).^5^ Incomplete outcome data (attrition bias).^6^ selective reporting (reporting bias).^7^ Other potential sources of bias. The reviewers judged the domains as: “low risk,” “high risk,” or “unclear.”

### 2.5 Data analysis

We used the RevMan software (5.4) to perform the meta‐analysis; the continuous outcomes were measured as mean difference (MD) and standard deviation (SD), and the dichotomous outcomes as risk ratios (RR) with 95% confidence interval (CI). In case of heterogeneity detected by the I‐square test over 50%, we used “leave‐one‐out” in general; the results were considered significant if the *p* < 0.05.

## 3 RESULTS

### 3.1 Study inclusion

**Figure 1:**
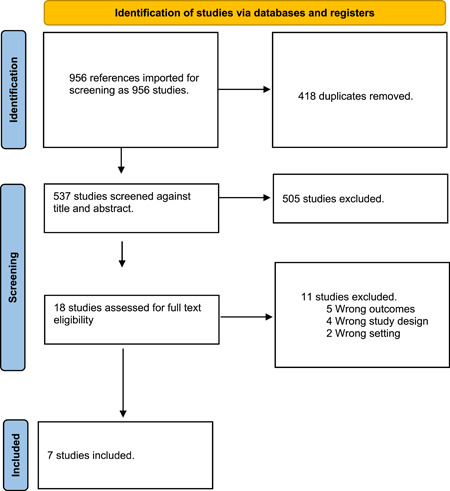
PRISMA flow diagram.

**Figure 2:**
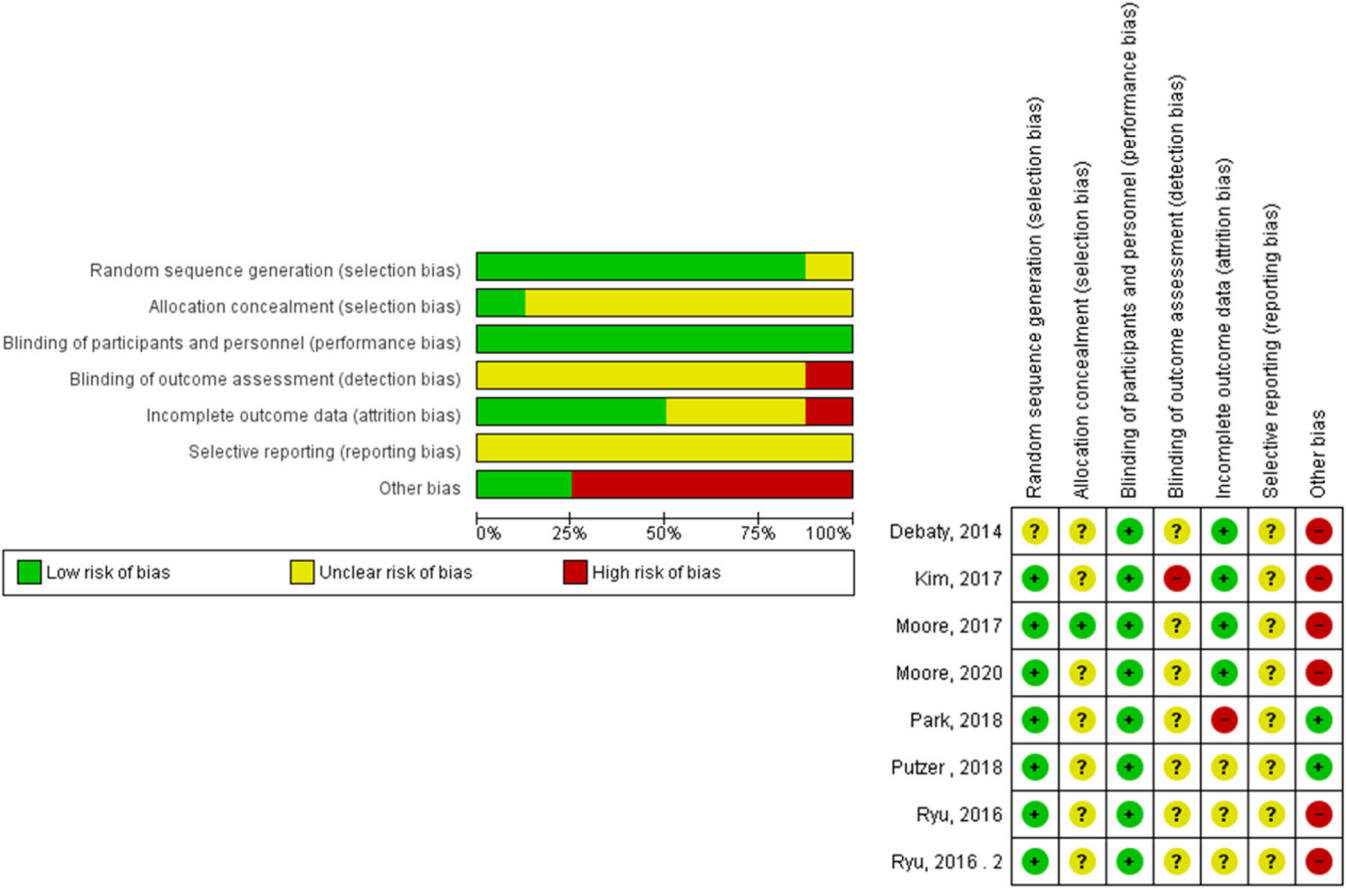
Risk of bias graph and assessment.

After a search of the literature, 956 publications resulted and then became 537 after the removal of duplicates. Of these, 18 were eligible for full‐text screening. After the full‐text screening, seven studies were included in our meta‐analysis, as shown in (Figure 1). Survival, cerebral flow, cerebral perfusion pressure, coronary perfusion pressure, systolic aortic pressure, and diastolic aortic pressure outcomes were reported in 3, 4, 4, 6, 5, and 6 studies, respectively. The “Ryu 2016” study was divided into two lessons because the trial was on two groups of pigs. The risk of bias assessment and summary of the included studies are shown in Figure 2 and Table S1, respectively. The overall bias was moderate in the included studies as we found bias in blinding of outcome assessment (detection bias) and incomplete outcome data (attrition bias) domains in only two studies and some other biases. We found no significant bias in random sequence generation (selection bias), allocation concealment (selection bias), blinding of participants and personnel (performance bias), and selective reporting (reporting bias).

### 3.2 Analysis

The total number of pigs included in the meta‐analysis in the heads‐up CPR group is 67 pigs, and the total number of pigs in the supine CPR group is 77 pigs.

**Figure 3:**
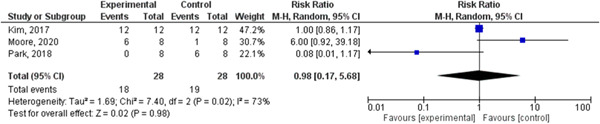
Forest plot of survival outcome.

**Figure 4:**
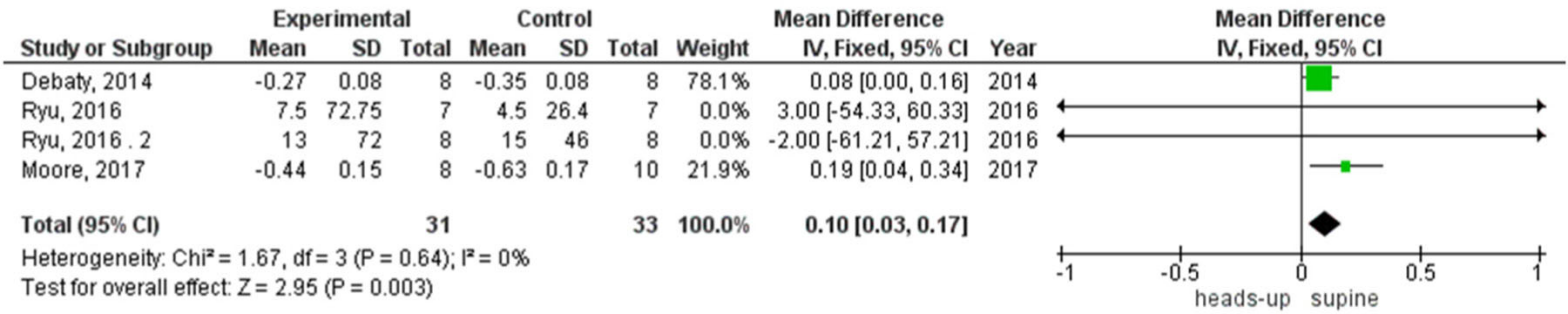
Forest plot of the cerebral flow outcome.

**Figure 5:**
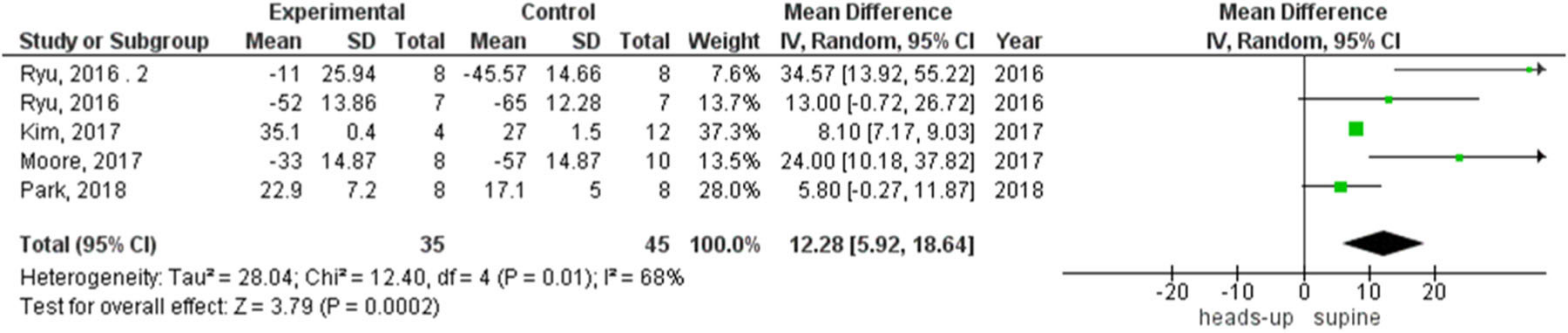
Forest plot of the cerebral perfusion pressure outcome.

**Figure 6:**
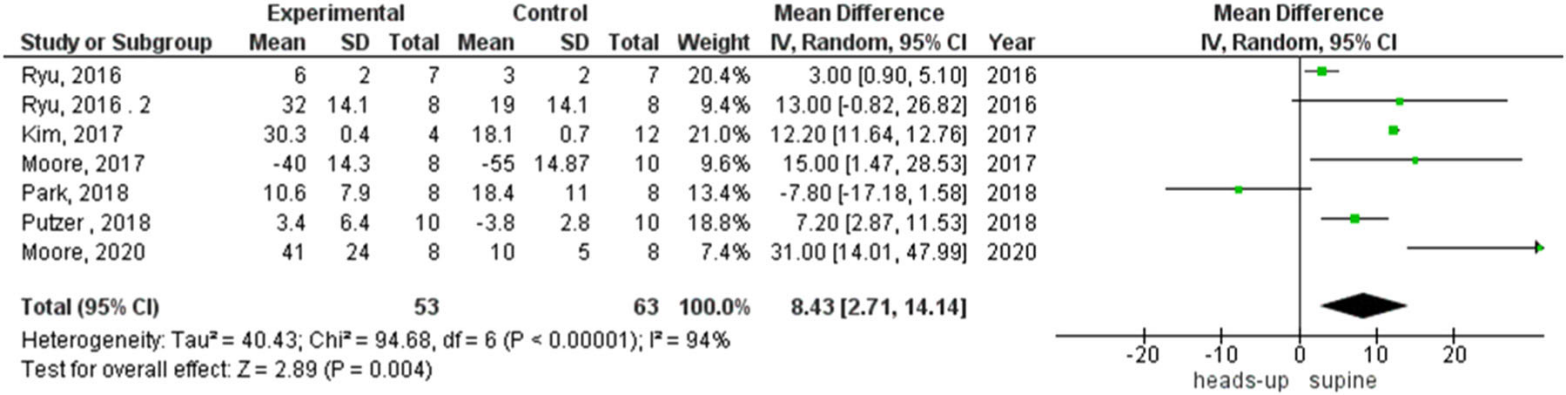
Forest plot of the coronary perfusion pressure outcome.

In survival outcome, the pooled analysis between heads‐up and supine CPR was (RR = 0.98, 95% CI = 0.17–5.68, *p* = 0.98), we observed heterogeneity that was not solved by leave‐one‐out test, as shown in Figure 3. The pooled analyses between heads‐up CPR and supine CPR in cerebral flow, cerebral perfusion pressure and coronary perfusion pressure outcomes, were (MD = 0.10, 95% CI = 0.03–0.17, *p* = 0.003), (MD = 12.28, 95% CI = 5.92–18.64, *p* = 0.0002) and (MD = 8.43, 95% CI = 2.71–14.14, *p* = 0.004), respectively (Figures 4, 5, 6, respectively).

**Figure 7:**
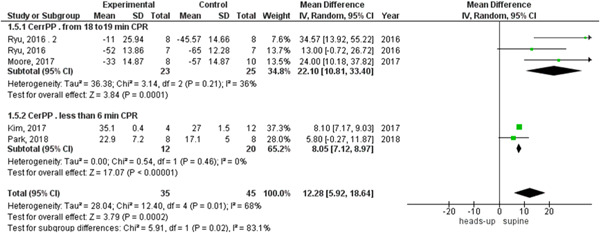
Forest plot of the cerebral perfusion pressure subgroup analysis.

We observed heterogeneity among studies that reported coronary perfusion pressure and cerebral perfusion pressure, which was not solved by random effects, so we did a subgroup analysis based on the duration of the CPR. The two subgroups were 6 min or less than 6 min CPR and from 18 to 20 min CPR, the heterogeneity was solved in each subgroup in the cerebral perfusion pressure outcome and the results were (MD = 8.05, 95% CI = 7.12–8.97, *p* < 0.00001) and (MD = 22.10, 95% CI = 10.81–33.40, *p* = 0.0001), respectively (Figure 7).

**Figure 8:**
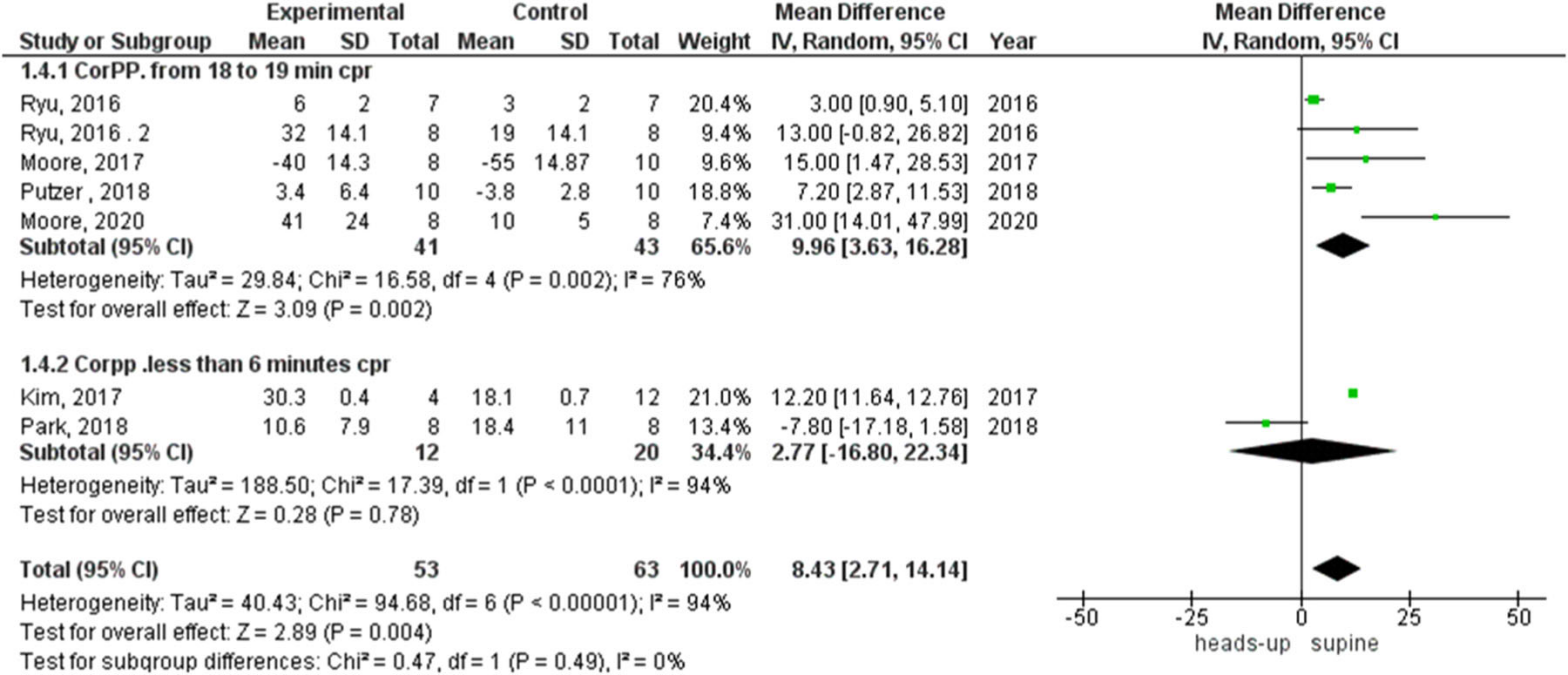
Forest plot of the coronary perfusion pressure subgroup analysis.

In the coronary perfusion pressure outcome, the heterogeneity was not solved by subgroup analysis or leave‐one‐out test. The results were in the 18‐ to 20‐min CPR subgroup (MD = 8.96, 95% CI = 3.63–16.28, *p* = 0.002) and in the subgroup of fewer than 6 min CPR were (MD = 2.77, 95% CI = −16.80 to 22.34, *p* = 0.78), as shown in Figure 8. There was no heterogeneity among studies that reported cerebral flow outcomes (Figure 4).

**Figure 9:**
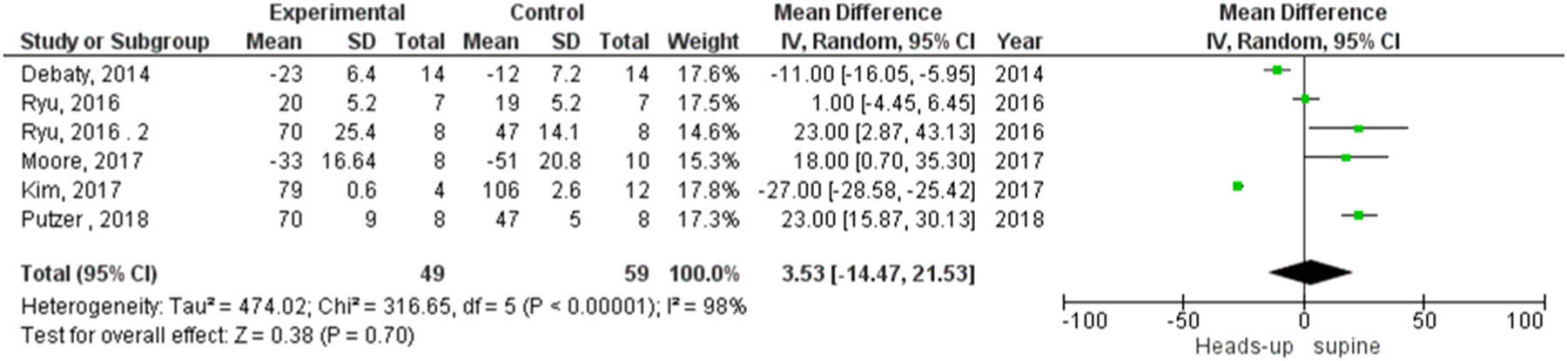
Forest plot of the systolic aortic pressure outcome.

**Figure 10:**
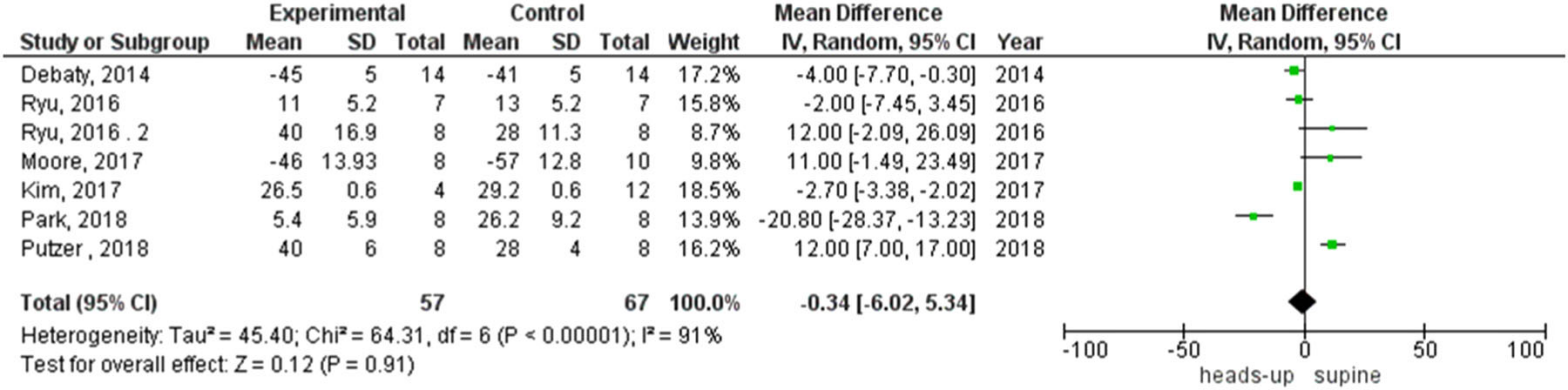
Forest plot of the diastolic aortic pressure outcome.

**Figure 11:**
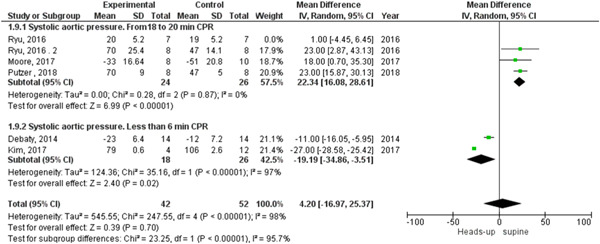
Forest plot of the systolic aortic pressure subgroup analysis with the leave‐one‐out test.

**Figure 12:**
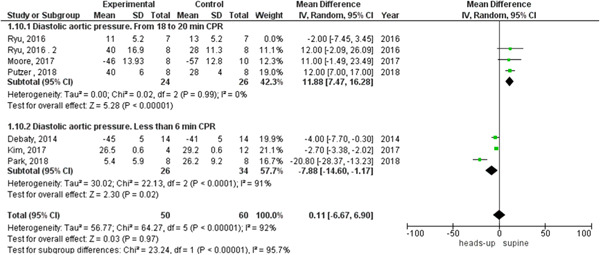
forest plot of the diastolic aortic pressure subgroup analysis with the leave‐one‐out test.

We found statistically significant heterogeneity among the studies that reported systolic and diastolic aortic pressure between heads‐up and supine CPR (Figures 9 and 10, respectively). In the systolic aortic pressure outcome, the heterogeneity was not solved by subgroup analysis and solved by performing leave‐one‐out a test by excluding (Ryu 2016 study) only in the subgroup of 18 to 20 min CPR and the results were in the 18‐ to 20‐min CPR subgroup (MD = 22.34, 95% CI = 16.08–28.61], *p* > 0.00001). In the subgroup of fewer than 6 min CPR, results were (MD = −19.19, 95% CI = − 34.86 to −3.51, *p* = 0.02), as shown in Figure 11. In the diastolic aortic pressure outcome, the heterogeneity was not solved by subgroup analysis and solved by doing a leave‐one‐out test by excluding (Ryu 2016 study) in the 18 to 20 min CPR subgroup only. The results were in the 18 to 20 min CPR subgroup (MD = 11.88, 95% CI = 7.47–16.28, *p* > 0.00001) and in the subgroup of fewer than 6 min CPR was (MD = −7.88, 95% CI = −14.60 to −1.17, *p* = 0.02) (Figure 12).

## 4 DISCUSSION

We found a statistically significant association between heads‐up CPR and increased cerebral flow, cerebral perfusion pressure, and coronary perfusion pressure compared with supine CPR. Subgroup analysis showed a statistically significant association between heads‐up CPR and increased cerebral perfusion pressure in subgroups of 6 min CPR, and from 18 to 20 min CPR compared with supine CPR. Subgroup analysis showed also a statistically significant association between heads‐up CPR and increased coronary perfusion pressure in a subgroup of 18 to 20 min CPR but no statistically significant difference between heads‐up CPR and supine CPR was detected in 6‐min CPR group. No statistically significant difference was found between heads‐up CPR and supine CPR positions in systolic and diastolic aortic pressure outcomes in the overall analysis. Subgroup analysis showed a statistically significant association between heads‐up CPR and increased systolic and diastolic aortic pressure outcomes in the subgroup of 18 to 20 min CPR, but in the subgroup of 6 min or less than 6 min CPR, we found a statistically significant association between heads‐up CPR and decreased systolic and diastolic aortic pressure outcomes compared with supine CPR. No statistically significant difference between heads‐up CPR and supine CPR was detected in the survival outcome.

### 4.1 The origin of heads‐up CPR and bundle approach

The conventional passive CPR method delivers a small percentage of baseline cardiac output, leading to inadequate perfusion of the heart and brain.^8^, ^9^ Due to these downfalls, Dr. Lurie developed a technique that increased venous return by elevating the chest during decompression intervals. This modification utilized direct contact via suction cups resulting in active compression–decompression (ACD) CPR.^10^ Additionally, elevating the head and chest of the patient during CPR increased blood flow to the brain while also increasing the venous runoff.^11^ Improved cerebral perfusion pressure (CerPP) was simply due to increased mean aortic pressure and decreased intracranial pressure (ICP) in the heads‐up CPR group.^11^ With head elevation, venous blood rapidly drains from the brain to the heart, reducing ICP and lowering the arterial and venous pressure waves.^12^

In the bundle approach of CPR, the synergistic effects of head and neck elevation result in the most significant improvement of cerebral and coronary perfusion pressures (CoPP).^13^ This new bundle approach showed its efficacy in improving rates of return of spontaneous circulation (ROSC) and sustained circulation when used in pre‐hospital cardiac arrest care plans.^5^ Greater rates of ROSC are associated with a higher coronary perfusion pressure value.^14^, ^15^

### 4.2 Heads‐up CPR experimental models

Our analysis compares heads‐up versus supine CPR techniques in seven studies including 144 pigs. Data reports improved cerebral perfusion with heads‐up CPR. Putzer et al.^16^ showed the effect of the prolonged CPR lasting 22 min on CerPP over time. At the end of the duration, the mean CerPP was shown to be below 4 mmHg which means nearly 7% of the original baseline CerPP in the head‐up group.^16^ Prolonged CerPP remained significantly higher in the heads‐up CPR pigs at 22 min than flat pigs in the ACD + threshold device (TD) and standard CPR groups.^16^ Notably, the CerPP was markedly higher in animals receiving heads‐up ACD + TD CPR compared with heads‐up standard CPR. No pigs treated with traditional CPR achieved successful resuscitation.^17^

Moore et al.^11^ implemented an experiment in pig models to assess the efficacy of heads‐up CPR on hemodynamics. They concluded that heads‐up CPR reduced ICP and improved cerebral perfusion which can be in part tied in with increased venous drainage from the head and paravertebral plexus.^11^

Moreover, there is conflicting evidence on the optimal degree of head elevation during heads‐up CPR. Venous pressures and ICP demonstrated a linear relationship with brain perfusion pressures. As venous pressures and ICP decreased, perfusion pressures increased. All parameters showed positive changes with each heads‐up angle elevation increase of 10° (increased CerPP, oxygenation, cerebral blood flow, decreased ICP, and venous pressures). Ng et al.^18^ found that ICP significantly reduced at 30° compared to the supine position, with cerebral perfusion pressure showing minimal elevation from 0° to 30°. Mean arterial pressure, global venous cerebral oxygenation along regional cerebral oxygenation remained consistent during the elevation of the head.^18^

On the contrary, Park et al.^19^ showed that heads‐up CPR in swine models was associated with decreased rates of ROSC compared to supine position. This was explained through hemodynamic compromise with heads‐up CPR. They concluded that decreased ICP is not necessarily associated with improved cerebral perfusion with subsequent poor ROSC achievement. Additionally, It has also been shown that there was a significant decrease in the blood flow within carotid circulation in stroke patients when placed in the heads‐up position.^20^, ^21^ This strengthens the argument that the utility of the heads‐up CPR technique should be on a case‐to‐case basis and not “one size fits all.” Thus, patients needing to be transferred, namely, cases that are taken from high‐rise buildings, need to be considered separately because heads‐up CPR may have possible adverse effects. When CPR was used in combination with the transfer sheet (supine), it provided a higher mean depth of compression than the 45° and 90° stretchers. Furthermore, the percentage of depth‐enough reduction was higher in the transfer sheet patients than in the 45° stretcher group.^20^, ^21^

Of note, this discordance might be partially attributed to different techniques applied during CPR. Park et al.^19^ utilized LUCAS 2 chest compression system with an impedance threshold device (ITD) of 10 cmH_2_O. They also started CPR after 15 min of untreated ventricular fibrillation. Rate of compressions was 100/min and the respiratory rate of 10/min.^19^ Moore et al.^11^ used another approach when they elicited untreated VF for 8 min followed by basic life support ACD CPR + ITD with 30:2 followed by advanced life support ACD CPR + ITD in both supine and heads‐up positions.^11^

Intriguingly, In the absence of ACD‐CPR, ITD, and automated CPR + ITD, heads‐up CPR alone failed in achieving the same levels of CerPP elevation as when utilized in adjunct.^13^, ^16^ When not used in combination with the whole‐body tilt positioning, the mean aortic pressure decreased during the initial automated CPR.^10^ With the head elevation in the bundle of controlled automated reperfusion of the whole body, providers observed a neurologic benefit despite 20 min of no‐flow time.^22^

Such conflicting findings demonstrate the need for more rigorous human trials to assess the utility and peak angles of cerebral blood flow, coronary blood flow, morbidity, and mortality. Such studies may lead to decreased morbidity and mortality in patients' post‐heads‐up CPR.

### 4.3 Downfalls of heads‐up CPR model

Safety and utility remain the primary concerns for the heads‐up CPR model. Knowing that, on average, CPR lasts over 20 min, studies performed assessing the head or head and torso elevation position versus supine in models of CPR for 20 min or under may correlate poorly to real life.^16^ A shortened CPR session in these models could ultimately show better outcomes in heads‐up CPR than in actuality. There is also the possibility of anatomical considerations causing poor results in the heads‐up tilt group. When cardiac arrest occurs, skeletal muscles such as the diaphragm are paralyzed, and gravity could easily pull down intrathoracic organs such as the heart and lung. Skeletal muscle paralysis in cardiac arrest patients could cause a shift in the contents of the thoracic cavity when placing the patients in the heads‐up position. This ultimately leads to an increased need for an approach to locate the head before compressions in heads‐up CPR, suggesting that the chest compression point could have changed when head‐up tilt CPR was performed.

It is reasonable to consider the approach of heads‐up CPR performance so that blood pooling in the extremities can be avoided by maintaining the vertical “uphill” aortic drive during elevation.^23^ However, preliminary findings seem to suggest the idea that the prone position for legs accompanied by head and torso elevation may be more ideal.^5^, ^24^ CPR performed in the whole‐body heads‐up over a long period precipitates dangerous venous blood pooling in the lower extremities by gravity,^16^ decreasing blood to the heart. It has been suggested that to overcome the venous pooling during heads‐up CPR; a tourniquet‐assisted device may be implemented to overcome the pooling.^25^ To improve the hemodynamics of patients in cardiac arrest, the performance of passive leg raising was shown to be unfavorable.^25^, ^26^

Neurological outcome improvements should be explored further in the context that increased sustained brain perfusion will ultimately lead to protection from reperfusion injury during CPR. Simply put, if the brain remains perfused, it would not need to get re‐perfused, lowering the potential brain damage. For example, a study showed that 11 of 25 pigs in the heads‐up group showed zero neurological impairment a week after CPR, whereas only 5 of the 20 in the supine model had the same result.^22^ Furthermore, brain injury arising in the arterial and venous blood vasculature pressure via each compression has also been decreased with head elevation.^13^, ^27^

The idea of “priming the pump” has also been suggested when attempting the heads‐up CPR technique with the ITD utilized where you first begin the CPR in the supine position before transferring the patient to the elevated heads‐up position.^22^, ^28^ The idea of elevation CPR “priming” has been further suggested to gradually increase the elevation level, again, with the utility of synchronized Use of the ITD.^11^, ^22^, ^23^ During the performance of CPR, when the head is placed in the heads‐up position, and the director is elevated too rapidly, it poses a danger because the aortic pressure may quickly decline due to gravity. Therefore, CPR should not be interrupted when the head is elevated.^23^

The most challenging downfall in heads‐up CPR remains the inability to experiment with these new techniques on humans and the reliability of animal modeling. Obviously, these studies cannot be performed in humans, and the ventricular fibrillation swine model is commonly used and accepted as the cardiac arrest model for animals.^29^ Importantly, pig anatomy is different from the human body. To establish the heads‐up tilt position for pigs, the upper limbs were stretched by ropes to allow the body to hang on the tilted table. These experimental methods did not conform to the normal physiology of the pig. In swine models, it has been shown that in the supine position, optimal chest compression point has varied between subjects.^28^ This possibly suggests the utility of electrocardiogram in CPR, rather than the lack of utility of heads‐up CPR, seeing it as a universal problem despite the position.

The sole elevation of the head model versus the head and torso elevation model has been debated. Exclusive elevation of the head was shown to increase CePP and CoPP,^13^, ^26^ drawing into question the added benefit or utility of elevating the torso. Additionally, heart placement based on body position should be taken into consideration. Heart position may not be optimal for establishing a compression point in the heads‐up position compared to the supine position.^25^

Although our analysis provides important evidence for future studies and for clinical application, our results were limited by the significant heterogeneity detected between studies which might be due to the minor differences in timing and techniques applied in the included studies. We used subgroup analysis to overcome these differences except for the level of elevation of the head (angle of elevation) which needs more data from future primary studies to determine the exact clinical effect of each degree of head level elevation. The small sample size is another limitation. Moreover, the included articles demonstrated some bias when assessed by the Cochrane Risk of Bias tool as we found bias in blinding of outcome assessment (detection bias) and incomplete outcome data (attrition bias) domains in only two studies and some other biases because most of the studies have no registered protocols. More research of higher quality is warranted before validating the heads‐up CPR technique for clinical practice.

## 5 CONCLUSION

We found a statistically significant association between increased cerebral flow and heads‐up CPR position, as compared to supine CPR position. However, no difference in survival was detected between the two techniques. More research is required to get conclusive evidence about the efficacy of heads‐up CPR compared to supine CPR positions in humans and to determine the effect of variables such as the automated device and level of head elevation on the outcomes of the CPR.

## AUTHOR CONTRIBUTIONS

**Joseph Varney**: conceptualization; methodology; project administration; supervision; validation; writing–original draft; writing–review & editing. **Karam R. Motawea**: conceptualization; formal analysis; methodology; project administration; supervision; validation; writing–original draft; writing–review & editing. **Mostafa Reda Mostafa**: data curation; writing–review & editing. **Yossef H. AbdelQadir**: data curation; methodology; writing–review & editing. **Merna Aboelenein**: data curation; methodology; writing–review & editing. **Omneya A. Kandil**: data curation; methodology; writing–review & editing. **Nancy Ibrahim**: data curation; methodology; writing–review & editing. **Hashim T. Hashim**: data curation; methodology. **Kimberly Murry**: data curation; methodology; writing–review & editing. **Garrett Jackson**: data curation; methodology; writing–review & editing. **Jaffer Shah**: data curation; methodology. **Maty Boury**: data curation; methodology; writing–review & editing. **Ahmed K. Awad**: data curation; methodology; writing–review & editing. **Priya Patel**: data curation; methodology; writing–review & editing. **Dina M. Awad**: data curation; writing–review & editing. **Samah S Rozan**: data curation; writing–review & editing. **Nesreen E. Talat**: data curation; writing–review & editing. All authors have read and approved the final version of the manuscript. Jaffer Shah had full access to all of the data in this study and takes complete responsibility for the integrity of the data and the accuracy of the data analysis.

## CONFLICTS OF INTEREST

The authors declare no conflicts of interest.

## TRANSPARENCY STATEMENT

Joseph Varney affirms that this manuscript is an honest, accurate, and transparent account of the study being reported; that no important aspects of the study have been omitted; and that any discrepancies from the study as planned (and, if relevant, registered) have been explained.

## Supporting information

Supporting Information

## Data Availability

The authors confirm that the data supporting the findings of this study are available within the article and/or its supplementary material.
