# Supplementary material for: Efficacy of heads‐up CPR compared to supine CPR positions: Systematic review and meta‐analysis
Source: Health Sci Rep. 2022 May 24;5(3):e644. doi: 10.1002/hsr2.644 (PMC9128396; doi:10.1002/hsr2.644)
Supplement: Supplementary file 1 — Supporting Information [file HSR2-5-e644-s001.docx]

| **ID** | **study region** | **Design** | **Setting of the intervention** | **Duration of CPR** | **Study Arms** | **Endpoints (outcomes)** | **Conclusion** |
| --- | --- | --- | --- | --- | --- | --- | --- |
| **Putzer ,2018** | Austria | experimental animal study | After 8 min of untreated CA external mechanical chest compression (LUCAS2TM, Physio Control, Redmond, WA, USA) with a compression depth of 52 mm, a compression rate of 102 min−1, Angle in head up position was 30 degrees | 20 minutes | 20 pigs but one excluded from the analysis randomized to either HUP (angle of elevation 30◦, 15 cm head elevation) n=10 or SUP n=9 | ICP was significantly lower in HUP compared to SUP animals after 5 min (18.0 ± 4.5 vs. 24.1 ± 5.2 mmHg; p=0.033) and 20 min (12.0 ± 3.4 vs. 17.8 ± 4.3 mmHg; p=0.023) of CPR. Accordingly, CPP was significantly higher in the HUP group after 5 min (11.2 ± 9.5 vs. 1.0 ± 9.2 mmHg; p=0.045) and 20 min (3.4 ± 6.4 vs.−3.8 ± 2.8 mmHg; p=0.023) of CPR. However, no difference was found in rSO2, PbtO2, ScvO2 and L/P ratio between groups after 20 min of CPR. | In this animal model of BLS CPR, HUP as compared to SUP did not improve cerebral oxygenation or metabolism. |
| **Moore,2020** | USA | experimental animal study | After 8 min of untreated VF, mechanical automatic compressions and impedance threshold device were attached and performed CPR at a rate of 30:2 compression. | 19 minutes | A total of 16 pigs were randomized to C-CPR group (n = 8) or ACD + ITD CSE group (n = 8). | In 16 animals, return of spontaneous circulation rate was 8/8 (100%) with ACD + ITD CSE and 3/8 (25%) for C-CPR (p = 0.026). For the primary outcome of neurologically intact survival, 6/8 (75%) pigs had a CPC score 1 or 2 with ACD + ITD CSE versus 1/8 (12.5%) with C-CPR (p = 0.04). Coronary perfusion pressure (mmHg, mean  SD) was higher with CSE at 18 min (41  24 versus 10  5, p = 0.004). rSO2 (%, mean  SD) and ETCO2 (mmHg, mean  SD) values were higher at 18 min with CSE (32  9 versus 17  2, p = 0.01, and 55 mmHg  10 versus 21 mmHg  4, p < 0.001), respectively. | The novel bundled resuscitation approach of CSE with ACD + ITD CPR increased favorable neurological survival versus C-CPR in a swine model of cardiac arrest. |
| **kim,2017** | korea | randomized experimental design | After 6 min of untreated VF, external mechanical chest compression was done using the LUCAS®2 Chest Compression System (Zolife AB, Lund, Sweden). The endotracheal tube was attached to an impedance threshold device with an opening pressure of −10 cmH2O. | 5 minutes | 12 pigs were randomly assigned to 1) head-up tilt (HUT) by three angles (30◦, 45◦, or 60◦) or 2) head-down tilt (HDT) by three angles (30◦, 45◦, or 60◦) and at 3) supine position between HUT and HDT positions. | With 60◦, 45◦, 30◦ head-down, 0◦ (supine), and 30◦, 45◦, 60◦ head-up positioning, mean(SD) CerPPs increased consistently as follows: 2.4(0.4), 9.3(1.6), 16.5(1.6), 27.0(1.5), 35.1(0.4), 39.4(0.6), and 39.9(0.3) mmHg, respectively. CorPPs were followings according to same angle: 12.9(2.5), 13.3(2.5), 12.8(0.4), 18.1(0.7), 30.3(0.4), 24.1(0.6), and 26.5(0.9) mmHg, respectively. The CerPPs were peak at HUT(45◦) and HUT(60◦), but CorPP was peak in HUT(30◦) and higher than HUT(45◦) and HUT(60◦). | Cerebral perfusion pressure during mechanical CPR were similar and highest in the HUT(45◦ and 60◦) positions whereas the peak coronary perfusion pressure was observed with HUT(30◦). |
| **Moore,2017** | USA | experimental animal study | After 8 min of VF active compression device and impedance threshold (Pneumatic Compression Controller;Ambu International, Glostrup, Denmark) was used to deliver compressions at rate of 80 compressions/min, with a 50% duty cycle. | 18 minutes | 18 pigs randomized to CPR in HUP (level of head elevation was 25 cm) (n = 8) or SUP (n = 10) | The mean blood flow (ml/min/g, mean ± SD) to the brain after 15 min of CPR was 0.42 ± 0.05 in the HUP group (n = 8) and 0.21 ± 0.04 SUP (n = 10), respectively, (p < 0.01). The HUP group also had statistically significantly lower intracranial pressures and higher calculated cerebral perfusion pressures after 5, 15, 19 (before adrenaline) and 20 (after adrenaline) minutes of HUT versus SUP CPR. | After prolonged ACD-CPR + ITD in the HUP position, brain blood flow was 2-fold higher versus the SUP position. These positive findings provide strong pre-clinical support to proceed with a clinical evaluation of elevation of the head and thorax during ACD-CPR + ITD in humans in cardiac arrest. |
| **Park,2018** | korea | experimental animal study | After 15 min of untreated VF, external mechanical chest compression was done using the automated LUCAS 2 Chest Compression System device at a compression rate of 100 times/minute. An impedance threshold device was attached to the endotracheal tube. | 6 minutes | initially included 18 but 2 were excluded and due to difference n survival rates it was terminated thus only sixteen were included as Head-up tilt position (n=8) and  Supine position (n=8) | In the 8 pigs from the head-up tilt position group, one showed return of spontaneous circulation (ROSC); all eight pigs expired within 24 hours. In the eight pigs from the supine position group, six had the ROSC; six pigs survived for 24 hours and two expired. The head-up position group showed lower 24-hour survival rate and lower ROSC rate than supine position group (P<0.01). | The use of head-up tilt position with 30 degrees during CPR showed lower 24-hour survival than the supine position. |
| **Ryu,2016** | korea | experimental animal study | After 8 min of VF active compression device and impedance threshold (Pneumatic Compression Controller;Ambu International, Glostrup, Denmark) was used to deliver compressions at rate of 100 compressions/min, with a 50% duty cycle. | 6 minutes | 30 pigs into 2 groups : Group A, pigs were randomized after 2 minutes of flat automated conventional (C) CPR to HUP(level of head elevation was 15 cm) (n = 7) or SUP (n = 7) C-CPR. In Group B, pigs were randomized after 2 minutes of automated active compression decompression (ACD) CPR plus an impedance threshold device (ITD) SUP CPR to either HUP (n = 8) or SUP (n = 8). | The primary outcome of the study was difference in CerPP (mmHg) between the HUP and SUP positions within groups. After 22 minutes of CPR, CerPP was 6 ± 3 mmHg in the HUP versus -5 ± 3 in the SUP (p = 0.016) in Group A, and 51 ± 8 versus 20 ± 5 (p = 0.006) in Group B. Coronary perfusion pressures after 22 minutes were HUP 6 ± 2 vs SUP 3 ± 2 (p = 0.283) in Group A and HUP 32 ± 5 vs SUP 19 ± 5, (p = 0.074) in Group B. In Group B, 6/8 pigs were resuscitated in both positions. No pigs were resuscitated in Group A. | The HUP position in both C-CPR and ACD + ITD CPR significantly improved CerPP. This simple maneuver has the potential to improve neurological outcomes after cardiac arrest. |
| **Debaty ,2014** | USA | experimental animal study | After 6 min of untreated VF, external mechanical chest compression was done using the automated LUCAS 1 Chest Compression System device at a compression rate of 100 times/minute. An impedance threshold device was attached to the endotracheal tube. | 5 minutes | 14 pigs with L-CPR + ITD in a 0◦ supine position for 3 min and 8 pigs assigned to 0◦, 10◦, 20◦,30◦, 40◦, 50◦head up tilt position. | Coronary perfusion pressure was 19 ± 2 mmHg at 0◦ vs. 30 ± 3 at 30◦ HUT (p < 0.001) and 10 ± 3 at 30◦ HDT (p < 0.001). Cerebral perfusion pressure was 19 ± 3 at 0◦ vs. 35 ± 3 at 30◦ HUT (p < 0.001) and 4 ± 4 at 30◦ HDT (p < 0.001). Brain–blood flow was 0.19 ± 0.04 ml min−1 g−1 at 0◦ vs. 0.27 ± 0.04 at 30◦ HUT (p = 0.01) and 0.14 ± 0.06 at 30◦ HDT (p = 0.16). Heart blood flow was not significantly different between interventions. With 0, 10, 20, 30, 40 and 50◦ HUT, ICP values were 21 ± 2, 16 ± 2, 10 ± 2, 5 ± 2, 0 ± 2, −5 ± 2 respectively, (p < 0.001), CerPP increased linearly (p = 0.001), and CPP remained constant | The HUP position in both C-CPR and ACD + ITD CPR significantly improved CerPP. This simple maneuver has the potential to improve neurological outcomes after cardiac arrest. |

**Abbreviations: (CA: cardiac arrest), (VF: ventricular fibrillation), (ACD: active compression decompression), (ITD: impedence threshold device), (CSE: controlled sequential elevation)**
